# Reduction of Deuterium Level Supports Resistance of Neurons to Glucose Deprivation and Hypoxia: Study in Cultures of Neurons and on Animals

**DOI:** 10.3390/molecules27010243

**Published:** 2021-12-31

**Authors:** Alexandr Kravtsov, Stanislav Kozin, Alexandr Basov, Elena Butina, Mikhail Baryshev, Vadim Malyshko, Arkady Moiseev, Anna Elkina, Stepan Dzhimak

**Affiliations:** 1 Department of Radiophysics and Nanothechnology, Physics Faculty, Kuban State University, 350040 Krasnodar, Russia; aakravtsov@mail.ru (A.K.); stas.fizika@list.ru (S.K.); son_sunytch79@mail.ru (A.B.); baryshev_mg@mail.ru (M.B.); anna013194@mail.ru (A.E.); 2 South Scientific Center of the Russian Academy of Sciences, Laboratory of Problems of Stable Isotope Spreading in Living Systems, 344006 Rostov-on-Don, Russia; Intro-2@rambler.ru; 3 Department of Fundamental and Clinical Biochemistry, Kuban State Medical University, 350063 Krasnodar, Russia; 4 Department of Technology of Fats, Cosmetics, Commodity Science, Processes and Devices, Kuban State Technological University, 350072 Krasnodar, Russia; butina_elena@mail.ru; 5 Department of Organization and Support of Scientific Activities, Kuban State Agrarian University, 350044 Krasnodar, Russia; moiseew_a@rambler.ru; 6 Department of Physics, K.G. Razumovsky Moscow State University of Technologies and Management (The First Cossack University), 109004 Moscow, Russia; 7 The V.M. Gorbatov Federal Research Center for Food Systems of the Russian Academy of Sciences, Experimental Clinic—Laboratory of Biologically Active Substances of Animal Origin, 109316 Moscow, Russia

**Keywords:** deuterium depleted water, hypoxia, neuron, rats

## Abstract

The effect of a reduced deuterium (D) content in the incubation medium on the survival of cultured neurons in vitro and under glucose deprivation was studied. In addition, we studied the effect of a decrease in the deuterium content in the rat brain on oxidative processes in the nervous tissue, its antioxidant protection, and training of rats in the T-shaped maze test under hypoxic conditions. For experiments with cultures of neurons, 7–8-day cultures of cerebellar neurons were used. Determination of the rate of neuronal death in cultures was carried out using propidium iodide. Acute hypoxia with hypercapnia was simulated in rats by placing them in sealed vessels with a capacity of 1 L. The effect on oxidative processes in brain tissues was assessed by changes in the level of free radical oxidation and malondialdehyde. The effect on the antioxidant system of the brain was assessed by the activity of catalase. The study in the T-maze was carried out in accordance with the generally accepted methodology, the skill of alternating right-sided and left-sided loops on positive reinforcement was developed. This work has shown that a decrease in the deuterium content in the incubation medium to a level of −357‰ has a neuroprotective effect, increasing the survival rate of cultured neurons under glucose deprivation. When exposed to hypoxia, a preliminary decrease in the deuterium content in the rat brain to −261‰ prevents the development of oxidative stress in their nervous tissue and preserves the learning ability of animals in the T-shaped maze test at the level of the control group. A similar protective effect during the modification of the ^2^H/^1^H internal environment of the body by the consumption of DDW can potentially be used for the prevention of pathological conditions associated with the development of oxidative stress with damage to the central nervous system.

## 1. Introduction

One of the main characteristics of chemical elements is the ratio of protons and neutrons in the nucleus, in connection with which they exist in the form of several isotopes. If isotopes do not have the ability to spontaneously decompose, then they are called stable isotopes, and if they decay, then they are called radioisotopes. It is known that more than half of all chemical elements have from two to ten stable isotopes [1], for example, the hydrogen atom occurs in nature in the form of two stable isotopes: protium and deuterium. Protium contains no neutron at all in its nucleus, whereas deuterium has one neutron. As a result, these two atoms differ from each other in atomic mass almost twice, which is the largest difference in atomic masses between stable isotopes ever known in nature, including in living organisms. At the same time, the natural level of deuterium in water is about −37‰ [2], and in living organisms the level of deuterium usually corresponds to the natural level and can vary depending on the differences in the content of deuterium (δ^2^H) in the environment (local territorial, seasonal, characteristics of liquid consumption by living organisms, etc. [3,4,5]). Despite the relatively low content of deuterium in the body, the available data indicate that it, as a trace element, is necessary for the normal functioning of various cells. At the same time, even relatively small increases or decreases in its concentration affect physiological processes at all levels: molecular, cellular, tissue, organ and organism [6,7,8,9,10]. Many studies have shown that a decrease in the deuterium content supports the body’s defense systems, including nonspecific mechanisms [11,12,13,14].

Moreover, it has now been established that the consumption of deuterium-depleted water (DDW) by animals, leading to a decrease in the deuterium content in the body, may have important consequences for the mechanisms that control long-term memory [15] and modulate the level of anxiety [16]. Epidemiological studies have shown that the content of deuterium in water can influence the frequency of affective disorders and depression [17].

At the same time, even a relatively small decrease in the level of deuterium in the body causes changes in the functioning of the nervous system [15,16,17]; nevertheless, to the best of our knowledge, recently only a few studies have considered the impact of deuterium on organisms representing different stages of organization of living matter. There is also a limited number of studies showing the exact level of deuterium in the body at which stimulatory or inhibitory effects are observed [11,18]. In general, such works are of interest for understanding the features of the course of certain physiological processes at different levels of deuterium, as well as their effect on the body as a whole and its resistance to stressful influences of various nature.

The aim of this work was to comprehensively study the effect of a reduced deuterium content at the cellular level on cultured neurons during glucose deprivation, as well as to assess the pro-oxidant-antioxidant balance of the brain tissue and learning at the organismal level in rats exposed to acute hypoxia.

## 2. Material and Methods

### 2.1. Obtaining Water with Different Deuterium Content

We used DDW containing δ^2^H = −679‰, produced on a set-up developed at Kuban State University [19,20]. Water that was physiologically complete in terms of mineral components and with a deuterium content of −679‰ was obtained by adding mineral salts to it (mineralization 314–382 mg/L: hydrocarbonates—144–180, sulfates <1, chlorides—60–76, calcium—6, magnesium—3, sodium—50–58, potassium—50–58). As a comparison, we used drinking water with a natural concentration of deuterium (δ^2^H = −37‰) with exactly the same mineralization.

### 2.2. Investigation of the Effect of Reduced Deuterium Content at the Cellular Level

#### 2.2.1. Chemicals and Supplements

Chemicals and additives were purchased from Sigma-Aldrich (St. Louis, MO, USA): propidium iodide, HEPES, poly-L-lysine, thiobarbituric acid; PanEco (Moscow, Russia): Minimum Essential Medium, fetal calf serum, glutamine, salts for solutions and culture media.

#### 2.2.2. Obtaining Neuronal-Glial Cell Cultures

For experiments with neurons in vitro we used 7–8-day-old cerebellar neuronal-glial cell cultures obtained by the method of enzymatic-mechanical dissociation from the brain of 7–9-day-old Wistar rats as described in article [21] with some modifications. After sacrificing the pups with ethyl ether, they were treated with 70% ethyl alcohol. After that, the brain was removed and placed on the horizontal surface of the Maximov chamber. Next, the brain was washed with Ca^2+^- and Mg^2+^-free PBS and the cerebellum was removed. Then the cerebellum was transferred into the well of the Maximov chamber containing 2–3 mL of PBS and excised with a scalpel. The tissues were exposed for 20 min to trypsin (0.05%) and EDTA (0.02%) dissolved in PBS at 36.5 °C. After that, the tissue was washed three times with PBS and once in the culture medium and then subjected to mechanical dissociation in the culture medium, of the following composition: 90% Minimum Essential Medium, 10% fetal calf serum, 2 mM glutamine, 10 mM HEPES buffer, 5 mM KCl. The cell suspension was centrifuged at 1500 rpm for 1 min. After that, the supernatant was removed, and the precipitate was resuspended in the nutrition medium with 25 mM KCl necessary for survival of the cerebellum granular neurons. The cultures were grown in 96-well plates coated with poly-L-lysine. Each well was added with 0.1 mL cell suspension at final density of 3–5 × 10^3^ cell/ mm^2^. The cells were cultured in a CO_2_ incubator at 36.5 °C and 98% relative humidity.

#### 2.2.3. Modeling Glucose Deprivation

The cultures were washed and incubated for two hours at a temperature of 36 °C in an incubation medium (IM) containing (in mM): NaCl—154, KCl—25, CaCl_2_—2,3, MgCl_2_—1, NaHCO_3_—3,6, Na_2_HPO_4_—0.35, HEPES—10, glucose—5.6. In total, two IM versions were prepared (differing in the deuterium content, δ^2^H): −37‰, −357‰. Then some of the cultures were placed into IM without glucose, having previously washed them with this medium. After 1 h, the glucose-free IM was replaced by the IM with glucose. The rest of the cultures were similarly manipulated without changing the glucose level. Then the cultures were placed in a thermostat at 36 °C for 24 h.

#### 2.2.4. Assessment of the Level of Neuronal Death

To determine the rate of neuronal death in cultures, propidium iodide was used. This probe is not permeable to membranes but penetrates damaged cells. By binding to DNA, it produces fluorescence with maximum excitation at 535 nm and emission at 617 nm. Propidium iodide was added to cultures at a concentration of 5 μg/mL for 15 min. Then, after washing out the dye three times, fluorescence was measured at an excitation wavelength of 535 nm, at an emission wavelength of 625 nm on a Filter Max F5 multifunctional microplate reader (Molecular Devices, San Jose, CA, USA) [22]. To avoid the influence of different deuterium content in the solution on the measurement results, loading the probe, washing and measurement were carried out in a solution with the same deuterium content. The measurement results were presented as a percentage; the fluorescence intensity of the control cultures at δ^2^H = −37‰ deuterium was taken as 100%. Data obtained in three-five independent experiments were used for the analysis.

### 2.3. Study of the Effect of Reduced Deuterium Content in Rats

#### 2.3.1. Animal Model

The animals were kept in a vivarium under natural light, with free access to food and water. The experiments were carried out in accordance with the requirements of the “Rules for works with experimental animals” (Order No. 708n of the Ministry of Health and Social Development of the Russian Federation dated 23 August 2010 “On approval of laboratory practice rules”), good laboratory practices (GLP), the Declaration of Helsinki (2000) and European Union Directive 2010/63/EU.

The experiment was carried out on 56 male Wistar rats aged 2.5 months (weighing from 240 to 270 g). The effect of DDW on the functional state of the central nervous system (CNS), pro-oxidant and antioxidant factors in the rat brain tissues was determined under normoxia (without hypoxia) and 1 day after hypoxic exposure.

#### 2.3.2. Simulation of Hypoxic Effects on Rats

Acute hypoxia with hypercapnia was simulated in rats by placing them in sealed vessels with a capacity of 1 L. The animals were kept in such conditions until the appearance of the first agonal sigh. Thereafter, the rats were removed and placed in standard cages.

The experiments were carried out in the first half of daylight hours. The animals were divided into four groups of 14 animals each:

- group 1 (NW, Control)—intact rats that received in the diet water with a deuterium concentration equal to the natural one (δ^2^H = −37‰) for six weeks, without hypoxic exposure;
- group 2 (DDW)—rats fed with DDW in the diet (δ^2^H = −679‰) for six weeks, without hypoxic exposure;
- group 3 (NW, Hypoxia)—rats fed with water with a deuterium concentration equal to the natural one (δ^2^H = −37‰) for six weeks, on the 43rd day of the experiment subjected to acute hypoxia;
- group 4 (DDW, Hypoxia)—rats that received DDW in the diet (δ^2^H = −679‰) for six weeks, on the 43rd day of the experiment subjected to acute hypoxia.

From each group, half of the animals participated in behavioral tests, the rest were decapitated in order to conduct biochemical studies of the brain.

#### 2.3.3. Study of Oxidative and Antioxidant Factors in the Rat Brain

The effect on oxidative processes in brain tissues was assessed by changes in the level of free radical oxidation (FRO) and malondialdehyde (MDA), one of the products of lipid peroxidation (LPO). The level of FRO was studied by chemiluminescence analysis on a SmartLum 5773 (DISoft, Moscow, Russia) device, which was used to determine the light sum of chemiluminescence, expressed in arbitrary units. The intensity of chemiluminescence was used to judge the content of free radicals in the brain tissues [23]. The MDA content was determined from the concentration of the colored complex formed during the interaction of MDA with thiobarbituric acid [24]. The effect on the antioxidant system of the brain was assessed by the index of catalase activity, which was determined by the decrease in hydrogen peroxide in the incubation medium [25].

#### 2.3.4. Study of the Behavior of Rats in the T-maze Test

The study in the T-maze was carried out in accordance with the generally accepted technique [26]: after accustoming the animals to the maze, the skill of alternating right-sided and left-sided loops on positive reinforcement was developed. To increase motivation, training was carried out against the background of food deprivation: rats subjected to 48-h food deprivation were placed in the starting compartment of the T-maze, in one of the arms of which there was a feeding trough with food. Thirty seconds after the rats were seated, the door of the starting compartment was opened. In this case, the click of the door opening served as a conditioned stimulus. The following indicators were taken into account: the percentage of correct choices of the labyrinth sleeve, the time of the conditioned reflex reaction. During 4 days of training, to develop the skill of bilateral alternation of right-sided and left-sided loops, each animal made 20 loops daily. Then, after 2 days, the preservation of the memorial trace was checked. The running data were averaged for each day of the experiment.

#### 2.3.5. Determination of Deuterium Content in the Brain of Rats

Brain samples were lyophilized in a vacuum freeze dryer “LS-1000” (Prointech, Pushchino, Russian Federation). Laboratory animals’ lyophilized brain isotope composition was determined on a DELTAplus mass spectrometer (Finnigan, Bremen, Germany) [27]. Solid samples were folded in a metallic foil and placed in the combustion chamber using a CARLO ERBA AS 200-LS (CARLO ERBA, Emmendingen, Germany) automatic sampler. The measurements in the solid-state samples were calibrated with respect to an international sample (IAEA-CH-7; δD = −100.3‰) and different internal verified standards. The data in the article are presented in ppm (‰).

### 2.4. Statistical Analysis

Statistical processing of the data obtained was carried out using STATISTICA 10. The reliability of the differences in the mean values (M) found between the groups was statistically evaluated using a nonparametric U-test (Mann–Whitney), the difference was considered reliable for *p* < 0.05. Data are presented as M ± m, where «M» is the mean, «m» is the standard error of the mean.

## 3. Results

### Influence of Environment with Different Deuterium Content on Neuronal Death during Glucose Deprivation

**Figure 1:**
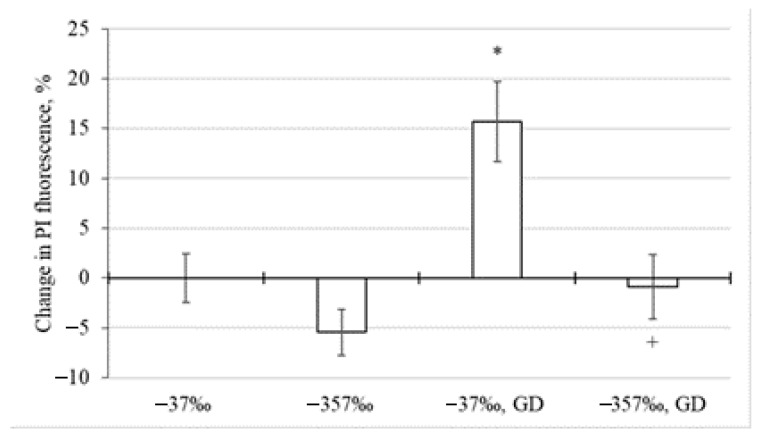
Changes in the neuronal death rate in the cerebellar neuronal-glial cell cultures during glucose deprivation (GD) depending on the concentration of deuterium in the incubation medium (δ^2^H = −37‰, −357‰), determined by the fluorescence of propidium iodide (PI). Data are presented as M ± m. *—*p* < 0.05 o control (δ^2^H = −37‰), +—*p* < 0.05 к δ^2^H = −357‰ with GD.

As the analysis of the experimental results showed, at δ^2^H of the incubation medium equal to −37‰, glucose deprivation led to an increase in the fluorescence intensity of propidium iodide by 16% in relation to the control value (Figure 1). A decrease in the deuterium content in the incubation medium to δ^2^H equal to −357‰ led to the maintenance of the probe fluorescence intensity at the level of the control value. In the absence of glucose deprivation at δ^2^H equal to −357‰, this indicator decreased by 5% (*p* ≥ 0.05). All this generally indicates that a decrease in the level of deuterium in the incubation medium to δ^2^H equal to −357‰ has a protective effect in glucose deprivation, manifested in the preservation of the neuronal death rate at the level of that for control cultures.

A study of the deuterium content in the rat brain showed a significant decrease in the deuterium content in the lyophilized brain tissue in animals that consumed DDW. So, if in the brain of control animals δ^2^H was −62‰, then in rats that consumed DDW, δ^2^H = −261‰. It should be noted that we used DDW containing δ^2^H = −679‰, since the use of water with δ^2^H = −357‰ while animals are continuing to consume food with natural levels of deuterium would not contribute to a sufficient decrease in the content of deuterium in body tissues.

**Figure 2:**
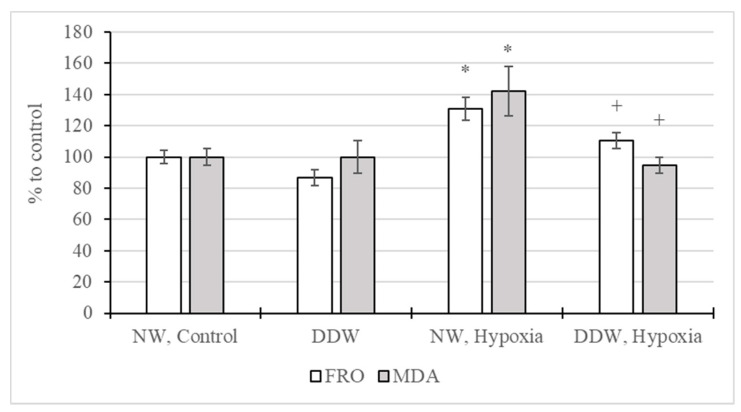
The intensity of free radical oxidation (FRO) and the content of malondialdehyde (MDA) in the brains of rats consuming normal water (NW) and deuterium-depleted water (DDW) under exposure to hypoxia (M ± m). *—*p* < 0.05 in comparison with control, +—*p* < 0.05 in comparison with “NW, Hypoxia”.

A study of the effect of DDW consumption on oxidative processes in the rat brain under acute hypoxia showed (Figure 2) that hypoxic exposure led to an increase in the intensity of free radical oxidation by 31%, as well as the MDA content by 42%. On the other hand, in the brains of animals that consumed DDW, these indicators remained at the level of the physiological norm and were significantly lower than in the group of hypoxified rats that received water with a natural level of deuterium. In the absence of hypoxia, a significant effect of DDW consumption on the FRO intensity and MDA concentration in the brain of rats has not been established.

**Figure 3:**
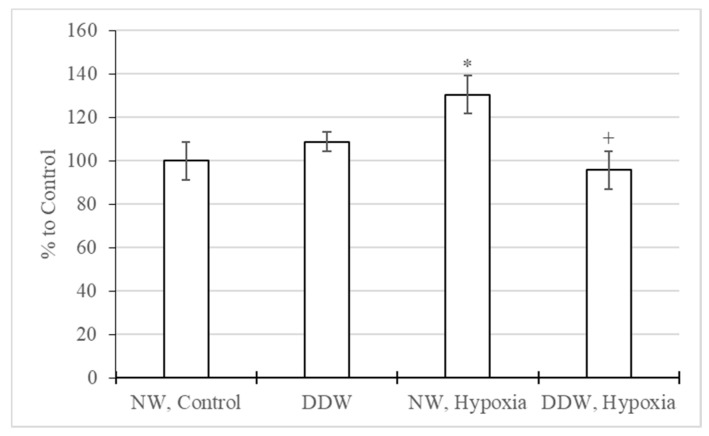
Catalase activity in the brain of rats consuming normal water (NW) and deuterium-depleted water (DDW) when exposed to hypoxia (M ± m). *—*p* < 0.05 compared with the “NW, Control” group, +—*p* < 0.05 compared with “NW, Hypoxia”.

It was also revealed that in rats exposed to hypoxic exposure and receiving water with a natural level of deuterium, catalase activity increased by 30% (Figure 3). Rats receiving DDW both with and without hypoxic exposure did not show statistically significant differences in catalase activity compared to the control group.

**Figure 4:**
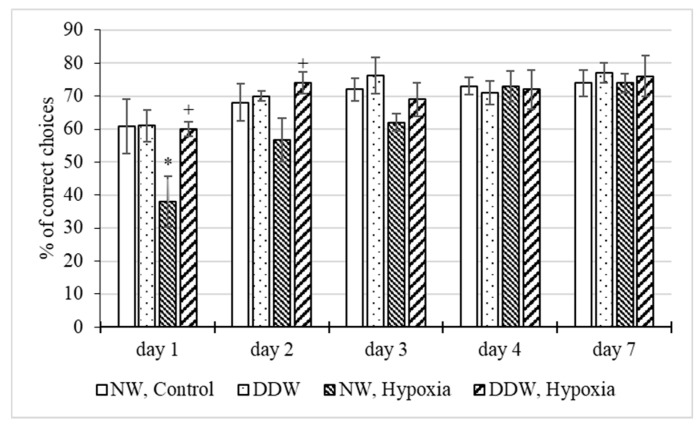
Influence of water with a low deuterium content (δ^2^H = −679‰) in the norm and under the influence of hypoxia on the % of correct choices in the “T-maze” test (M ± m). *—*p* < 0.05 in comparison with the “NW, Control” group, +—*p* < 0.05—in comparison with the “NW, Hypoxia” group.

A study of the effect of water with a low deuterium content on the development of the skill of correct alternation of sleeve in the T-maze with positive reinforcement in rats under normal and hypoxic conditions (Figure 4) showed that DDW does not normally affect the learning ability of animals. The number of correct entries into the baited sleeve in the “DDW” group on all training days practically did not differ from those in the control. The number of correct choices in animals “NW, Hypoxia” was significantly lower (by 22.9%) than in the control on the first day of training. On the second day, the percentage of correct elections in this group, although it increased to 56.7%, was still lower than in the control group (68.1%). The tendency to an increase in the learning ability of rats in the “NW, Hypoxia” group was noted in the following days. On the fourth day, the percentage of correct entries into the baited sleeve practically did not differ from the control. Meanwhile in the “DDW, Hypoxia” group the number of correct choices on all days of the study was at the control level, while on the first and second days it significantly exceeded that in the “NW, Hypoxia” group.

**Figure 5:**
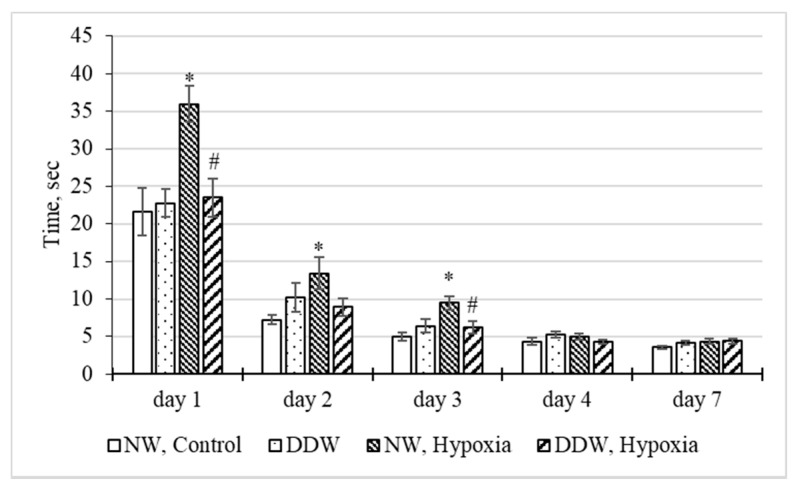
Influence of water with a low deuterium content (δ^2^H = −679‰) on the duration of the conditioned reflex reaction in the “T-maze” test (M ± m). *—*p* < 0.05, compared with the “NW, Control” group, #—*p* < 0.05—compared with the “NW, Hypoxia” group.

In addition, in the “NW, Hypoxia” group (Figure 5), there were marked differences in the timing of the performance of the conditioned reflex reaction (CRR). So, despite the fact that on the first day of training all animals had the longest decision-making time, in the “NW, Hypoxia” group it significantly exceeded (by 66.7%) that in the control group and by 52.7% in the group of rats that received water with a reduced deuterium content and were subjected to hypoxia (“DDW, Hypoxia”). Although the decision-making time was gradually decreasing every day in all groups, in the “NW, Hypoxia” group it was longer than in the control (from 1 to 3 days), and significantly longer than in the “DDW, Hypoxia” group (for 1 and 3 days). Only on the fourth day of training did the time for performing the CRR in all groups not practically differ from the “NW, Control” group.

Two days after the end of training, all animals retained the skills of conditioned reflex reaction of bilateral alternation of right- and left-sided loops, while there were no significant differences between the groups. There were no differences in the timing of the conditioned reflex reaction. Thus, it was found that long-term use of DDW in the absence of hypoxia does not affect the learning process of rats in the T-maze test, while its preventive use before hypoxic exposure helps to maintain the learning ability of rats at the level of natural indicators of the control group (“NW, Control”).

## 4. Discussion

It is known that deficiency in the amount of oxygen and glucose reaching the neural tissues during hypoxia/ischemia induces a chain of sequential reactions that trigger the processes of neuronal damage and cell death [28,29]. Thus, energy deficiency leads to the failure of energy-dependent ion pumps, resulting in the release of toxic concentrations of excitatory neurotransmitters such as glutamate [30]. The increasing influx of calcium into the cytoplasm of the neuron leads to the activation of a number of intracellular signaling cascades leading to the overproduction of reactive oxygen species and other free radicals [31,32]. In particular, this is facilitated by mitochondrial depolarization, activation of cytosolic phospholipase A2, catalysis of arachidonic acid by cyclooxygenase-2, and activation of NADPH oxidase [33]. The formation of free radicals is further accelerated upon restoration of oxygen supply after reoxygenation/reperfusion, further enhancing lipid peroxidation, protein oxidation, and DNA damage [34]. In parallel, the depletion of the antioxidant resources of the cell occurs, which leads to the development of oxidative stress [35].

On the other hand, the overproduction of reactive oxygen species [36,37], and at the next stage, lipid peroxidation [38], is an integral part of the general “adaptation-stress” syndrome [39]. In this case, the activation of free radical processes also serves as a nonspecific signal for the mobilization of the body’s defense systems [40,41,42,43], including the activation of antioxidant defense mechanisms [44], which is a typical reaction of the biological system to an effect that unbalances it from the state of physiological equilibrium [45,46,47,48].

In our experiments, we found an increase in response to the hypoxic effect of free- radical oxidation and the accumulation of lipid peroxidation products (primarily MDA), as well as an increase in catalase activity in the brain tissue of rats that consumed water with a natural level of deuterium. On the other hand, all the studied parameters in the brain tissue of animals that consumed DDW remained at the level of the physiological norm, both during hypoxia and in its absence. Our results indicate that the consumption of DDW for six weeks increases the adaptive potential of the rat organism, which is manifested in the maintenance of the parameters of oxidative processes and antioxidant protection in the brain at the normal level when exposed to hypoxia.

The learning process consists of obtaining new information, processing it and consolidating it in behavioral responses and skills. Processing information entering the brain requires a high coordination of cellular activity in the cortical networks [49,50], as well as in the hippocampus. As is known, coordination in a neural network can be provided by oscillations in certain frequency ranges. Network oscillations are distinguished in theta (4–12 Hz), beta (13–30 Hz) and gamma (30–100 Hz) ranges, which are associated with various cognitive and behavioral functions [51]. Gamma oscillations (30–100 Hz) have been found in many areas of the mammalian brain, such as the visual, auditory, somatosensory and motor systems, as well as in the hippocampus. It has been established that gamma oscillations provide a fundamental mechanism of information processing during sensory perception, motor behavior, choice of an object of attention and memory formation by coordinating neuronal activity in the networks of the hippocampus and neocortex [52,53]. It is noted that gamma oscillations require a high energy consumption of neurons, which is due to the increased rates of action potentials and postsynaptic potentials. This is due to the fact that in order to maintain homeostasis and excitability of neurons, ionic gradients used to generate potentials must be constantly restored by ion pumps, such as Na^+^/K^+^-ATPase and Ca^2+^-ATPase, as well as carriers, such as Na^+^/Ca^2+^-exchanger. Na^+^/H^+^-exchanger and K^+^/Cl^−^-cotransporter [52,54,55,56,57,58]. In particular, the maintenance of K^+^homeostasis during gamma oscillations is apparently achieved due to a greatly increased generation of ATP, requires a proportionally high supply of oxygen and the functioning of oxidative phosphorylation systems with an intensity close to the limiting one [51]. The synthesis, release and absorption of neurotransmitters and their precursors also require energy resources [59,60,61,62,63]. In addition, neuronal activity stimulates abundant protein synthesis, which also requires significant energy inputs [64]. This testifies to the high energy consumption of the functioning of the rat brain, especially in the learning process.

As noted above, in the brain of rats that consumed water with natural levels of deuterium, hypoxic exposure induced oxidative stress. This means that part of the resources of the biological system, including energy components, was used to maintain the resource of the antioxidant system, as well as restore the balance of redox processes, transmembrane ion gradients, and repair damaged structures [65]. Thus, the energy resources of the rat brain in this group, which are so necessary for ensuring the higher functions of the central nervous system during the adaptation of their nervous tissue to hypoxia, were limited. In this regard, the significantly lower performance indicators observed in our experiments in the development of the skill of correct alternation of arms in the T-maze in rats of the “NW, Hypoxia” group can probably be caused by problems in providing the necessary ionic homeostasis required for the coordinated work of neural networks in the learning process. As a result, rats exposed to hypoxic exposure may experience difficulties in focusing attention, as well as in memorization.

Moreover, we found that the consumption of DDW (δ^2^H = −679‰) for six weeks lead to a decrease in the content of deuterium in the rat brain from δ^2^H equal to −62‰ to δ^2^H equal to −261‰. This fact indicates that the results obtained in biochemical tests, as well as in experiments in the T-maze, are stipulated precisely by the change in the concentration of deuterium. This is also consistent with the results of experiments on cerebellar cell cultures, which showed that placing neurons in a medium containing δ^2^H equal to −357‰ increases their resistance to glucose deprivation, which was used to model pathological processes in cerebral ischemia [66,67]. In this case, the response of cultured neurons is generally comparable to the response of brain cells in vivo, when intensification of free radicals’ synthesis, lipid peroxidation, and changes in the activity of antioxidant enzymes are observed [68,69]. It is known that glucose deprivation causes energetic starvation of neurons, which use it as the main resource for providing energy-consuming metabolic processes [70]. Since hypoxic exposure also causes energy hunger, but due to the lack of oxygen [71,72], it can be assumed that the mechanism providing an increase in the resistance of cultured neurons to glucose deprivation when placed in an environment with δ^2^H equal to −357‰ is largely relevant to the mechanism that is realized with a decrease in the level of deuterium (δ^2^H = −261‰) in the rat brain for its protection from hypoxia (in vivo). This is consistent with the hypothesis [73], suggesting that with a moderate decrease in deuterium levels, an increase in the membrane potential of mitochondria occurs, which also contributes to the maintenance of ATP production.

## 5. Conclusions

In general, our results indicate an increase in the adaptive resource of the rat brain with a decrease in the deuterium content in the brain to δ^2^H equal to −261‰, which under the influence of hypoxia is manifested by the absence of both excessive intensification of oxidative processes and imbalance in the functioning of antioxidant defense, and is also characterized by a preserved ability to learn of these rats compared to intact animals. The studies carried out also indicate similar mechanisms of neuroprotection realization under hypoxic conditions when rats consume DDW and an increase in neuron resistance during glucose deprivation under conditions of their cultivation in a medium with a low deuterium content. Considering the above, the occurrence of a similar protective effect upon modification of the ^2^H/^1^H internal environment of the body by the consumption of DDW can potentially be used for the prevention of pathological conditions associated with the development of oxidative stress in case of damage to the central nervous system, including the aim of increasing the learning efficiency of living organisms under conditions of exposure to adverse (stress) environmental factors.

## Data Availability

Not applicable.

## References

[1] Kselíková V., Vítová M., Bišová K. (2019). Deuterium and its impact on living organisms. Folia Microbiol..

[2] Basov A., Fedulova L., Baryshev M., Dzhimak S. (2019). Deuterium-depleted water influence on the isotope ^2^H/^1^H regulation in body and individual adaptation. Nutrients.

[3] Basov A., Fedulova L., Vasilevskaya E., Dzhimak S. (2019). Possible Mechanisms of Biological Effects Observed in Living Systems during ^2^H/^1^H Isotope Fractionation and Deuterium Interactions with Other Biogenic Isotopes. Molecules.

[4] Somlyai G., Somlyai I., Fórizs I., Czuppon G., Papp A., Molnár M. (2020). Effect of Systemic Subnormal Deuterium Level on Metabolic Syndrome Related and other Blood Parameters in Humans: A Preliminary Study. Molecules.

[5] Halenova T., Zlatskiy I., Syroeshkin A., Maximova T., Pleteneva T. (2020). Deuterium-Depleted Water as Adjuvant Therapeutic Agent for Treatment of Diet-Induced Obesity in Rats. Molecules.

[6] Elharram A., Czegledy N.M., Golod M., Milne G.L., Pollock E., Bennett B.M., Shchepinov M.S. (2017). Deuterium-reinforced polyunsaturated fatty acids improve cognition in a mouse model of sporadic Alzheimer’s disease. FEBS J..

[7] Fedulova L.V., Basov A.A., Vasilevskaya E.R., Dzhimak S.S. (2019). Gender difference response of male and female immunodeficiency rats treated with tissue-specific biomolecules. Curr. Pharm. Biotechnol..

[8] Andreyev A.Y., Tsui H.S., Milne G.L., Shmanai V.V., Bekish A.V., Fomich M.A., Pham M.N., Nong Y., Murphy A.N., Clarke C.F. (2015). Isotope-reinforced polyunsaturated fatty acids protect mitochondria from oxidative stress. Free Radic. Biol. Med..

[9] Yaglova N.V., Obernikhin S.S., Timokhina E.P., Yaglov V.V. (2021). Response of Pituitary-Thyroid Axis to a Short-Term Shift in Deuterium Content in the Body. Bull. Exp. Biol. Med..

[10] Basov A.A., Kozin S.V., Bikov I.M., Popov K.A., Moiseev A.V., Elkina A.A., Dzhimak S.S. (2019). Changes in prooxidant-antioxidant system indices in the blood and brain of rats with modelled acute hypoxia which consumed a deuterium-depleted drinking diet. Biol. Bull..

[11] Yaglova N.V., Obernikhin S.S., Timokhina E.P., Diatropova M.A., Diatropov M.E., Yaglov V.V. (2021). Impact of lower deuterium intake to the organism on thermoregulation. Bull. Exp. Biol. Med..

[12] Kozin S., Skrebitsky V., Kondratenko R., Kravtsov A., Butina E., Moiseev A., Malyshko V., Baryshev M., Elkina A., Dzhimak S. (2021). Electrophysiological activity and survival rate of rats nervous tissue cells depends on D/H isotopic composition of medium. Molecules.

[13] Beaudoin-Chabot C., Wang L., Smarun A.V., Vidovic D., Shchepinov M.S., Thibault G. (2019). Deuterated polyunsaturated fatty acids reduce oxidative stress and extend the lifespan of *C. elegans*. Front. Physiol..

[14] Basov A., Drobotenko M., Svidlov A., Gerasimenko E., Malyshko V., Elkina A., Baryshev M., Dzhimak S. (2020). Inequality in the Frequency of the Open States Occurrence Depends on Single ^2^H/^1^H Replacement in DNA. Molecules.

[15] Mladin C., Ciobica A., Lefter R., Popescu A., Bild W. (2014). Deuterium-depleted water has stimulating effects on long-term memory in rats. NeurosciLetter.

[16] Mladin C., Ciobica A., Lefter R., Popescu A., Bild W. (2014). Deuterium depletion induces anxiolytic-like effects in rats. Arch. Biol. Sci..

[17] Strekalova T., Evans M., Chernopiatko A., Couch Y., Costa-Nunes J., Cespuglio R., Chesson L., Vignisse J., Steinbusch H.W., Anthony D.C. (2015). Deuterium content of water increases depression susceptibility: The potential role of a serotonin-related mechanism. Behav. Brain Res..

[18] Podlesak D.W., Torregrossa A.-M., Ehleringer J.R., Dearing M.D., Passey B.H., Cerling T.E. (2008). Turnover of oxygen and hydrogen isotopes in the body water, CO_2_, hair, and enamel of a small mammal. Geochim. Cosmochim. Acta.

[19] Frolov V.Y., Baryshev M.G., Dzhimak S.S., Lomakina L.V., Bolotin S.N., Petriev I.S. (2013). A Method of Obtaining Water with a Reduced Deuterium. Content. Patent.

[20] Petriev I., Pushankina P., Lutsenko I., Shostak N., Baryshev M. (2020). Synthesis, Electrocatalytic and Gas Transport Characteristics of Pentagonally Structured Star-Shaped Nanocrystallites of Pd-Ag. Nanomaterials.

[21] Stelmashook E.V., Isaev N.K., Plotnikov E.Y., Uzbekov R.E., Alieva I.B., Arbeille B., Zorov D.B. (2009). Effect of transitory glucose deprivation on mitochondrial structure and functions in cultured cerebellar granule neurons. Neurosci. Lett..

[22] Lau A.C., Cui H., Tymianski M. (2007). The use of propidium iodide to assess excitotoxic neuronal death in primary mixed cortical cultures. Methods Mol. Biol..

[23] Farkhutdinov R.R., Likhovskikh V.A. (1995). Chemiluminescent Methods for the Study of Free Radical Oxidation in Biology and Medicine.

[24] Gavrilov V.B., Gavrilova A.R., Mazhul’ L.M. (1987). Analysis of methods for measuring lipid peroxidation products in blood serum by the reaction with thiobarbituric acid. Vopr. Med. Khimii..

[25] Korolyuk M.A., Ivanova L.I., Maiorova I.G., Tokarev V.E. (1988). Methods of measuring catalase activity. Lab. Delo.

[26] Shurygina L.V., Zlishcheva E.I., Kravtsova A.N., Kravtsov A.A. (2017). Antioxidant and Antiamnestic Effects of Potassium Comenate and Comenic Acid under Conditions of Normobaric Hypoxia with Hypercapnia. Bull. Exp. Biol. Med..

[27] Dzhimak S.S., Barishev M.G., Basov A.A., Timakov A.A. (2014). Influence of deuterium depleted water on freeze dried tissue isotopic composition and morphofunctional body performance in rats of different generations. Biophysics.

[28] Maffezzini C., Calvo-Garrido J., Wredenberg A., Freyer C. (2020). Metabolic regulation of neurodifferentiation in the adult brain. Cell. Mol. Life Sci..

[29] Fathollahipour S., Patil P.S., Leipzig N.D. (2018). Oxygen Regulation in Development: Lessons from Embryogenesis towards Tissue Engineering. Cells Tissues Organs..

[30] Prasad K.N., Bondy S.C. (2020). Increased oxidative stress, inflammation, and glutamate: Potential preventive and therapeutic targets for hearing disorders. Mech. Ageing Dev..

[31] Mukandala G., Tynan R., Lanigan S., O’Connor J.J. (2016). The Effects of Hypoxia and Inflammation on Synaptic Signaling in the CNS. Brain Sci..

[32] Minhas G., Mathur D., Ragavendrasamy B., Sharma N.K., Paanu V., Anand A. (2017). Hypoxia in CNS Pathologies: Emerging Role of miRNA-Based Neurotherapeutics and Yoga Based Alternative Therapies. Front. Neurosci..

[33] Sun M.S., Jin H., Sun X., Huang S., Zhang F.L., Guo Z.N., Yang Y. (2018). Free Radical Damage in Ischemia-Reperfusion Injury: An Obstacle in Acute Ischemic Stroke after Revascularization Therapy. Oxid. Med. Cell Longev..

[34] Paul S., Candelario-Jalil E. (2020). Emerging neuroprotective strategies for the treatment of ischemic stroke: An overview of clinical and preclinical studies. Exp. Neurol..

[35] Akyuva Y., Nazıroğlu M. (2020). Resveratrol attenuates hypoxia-induced neuronal cell death, inflammation and mitochondrial oxidative stress by modulation of TRPM2 channel. Sci. Rep..

[36] An Z., Yan J., Zhang Y., Pei R. (2020). Applications of nanomaterials for scavenging reactive oxygen species in the treatment of central nervous system diseases. J. Mater. Chem. B..

[37] Chen Y., Qin C., Huang J., Tang X., Liu C., Huang K., Xu J., Guo G., Tong A., Zhou L. (2020). The role of astrocytes in oxidative stress of central nervous system: A mixed blessing. Cell Prolif..

[38] Nuñez A., Benavente I., Blanco D., Boix H., Cabañas F., Chaffanel M., Fernández-Colomer B., Fernández-Lorenzo J.R., Loureiro B., Moral M.T. (2018). Oxidative stress in perinatal asphyxia and hypoxic-ischaemic encephalopathy. An. Pediatr..

[39] Zambuto S.G., Serrano J.F., Vilbert A.C., Lu Y., Harley B.A.C., Pedron S. (2020). Response of neuroglia to hypoxia-induced oxidative stress using enzymatically crosslinked hydrogels. MRS Commun..

[40] Bailey A.P., Koster G., Guillermier C., Hirst E.M., MacRae J.I., Lechene C.P., Postle A.D., Gould A.P. (2015). Antioxidant Role for Lipid Droplets in a Stem Cell Niche of Drosophila. Cell.

[41] Watts M.E., Pocock R., Claudianos C. (2018). Brain Energy and Oxygen Metabolism: Emerging Role in Normal Function and Disease. Front. Mol. Neurosci..

[42] Shukla S.K., King R.J., Singh P.K. (2018). Transcriptional profiling using RNA-Seq to study hypoxia-mediated gene regulation. Methods Mol. Biol..

[43] Radhakrishnan S., Martin C.A., Dhayanithy G., Reddy M.S., Rela M., Kalkura S.N., Sellathamby S. (2021). Hypoxic Preconditioning Induces Neuronal Differentiation of Infrapatellar Fat Pad Stem Cells through Epigenetic Alteration. ACS Chem. Neurosci..

[44] Merelli A., Repetto M., Lazarowski A., Auzmendi J. (2020). Hypoxia, Oxidative Stress, and Inflammation: Three Faces of Neurodegenerative Diseases. J. Alzheimers Dis..

[45] Terraneo L., Paroni R., Bianciardi P., Giallongo T., Carelli S., Gorio A., Samaja M. (2017). Brain adaptation to hypoxia and hyperoxia in mice. Redox. Biol..

[46] Bonkowsky J.L., Son J.H. (2018). Hypoxia and connectivity in the developing vertebrate nervous system. Dis. Model. Mech..

[47] Buxton R.B. (2021). The thermodynamics of thinking: Connections between neural activity, energy metabolism and blood flow. Phil. Trans. R Soc. B.

[48] Raberin A., Nader E., Lopez Ayerbe J., Alfonsi G., Mucci P., Rytz C.L., Pialoux V., Durand F. (2021). Pro-Oxidant/Antioxidant Balance during a Prolonged Exposure to Moderate Altitude in Athletes Exhibiting Exercise-Induced Hypoxemia at Sea-Level. Life.

[49] Drevets W.C. (2004). Neuroplasticity in mood disorders. Dialogues Clin. Neurosci..

[50] Zhao F., Yang J., Cui R. (2017). Effect of Hypoxic Injury in Mood Disorder. Neural. Plast..

[51] Kann O., Hollnagel J.O., Elzoheiry S., Schneider J. (2016). Energy and Potassium Ion Homeostasis during Gamma Oscillations. Front. Mol. Neurosci..

[52] Kann O., Papageorgiou I.E., Draguhn A. (2014). Highly energized inhibitory interneurons are a central element for information processing in cortical networks. J. Cereb. Blood Flow Metab..

[53] Khuu M.A., Pagan C.M., Nallamothu T., Hevner R.F., Hodge R.D., Ramirez J.M., Garcia A.J. (2019). Intermittent Hypoxia Disrupts Adult Neurogenesis and Synaptic Plasticity in the Dentate Gyrus. J. Neurosci..

[54] Somjen G.G. (2002). Ion regulation in the brain: Implications for pathophysiology. Neuroscientist.

[55] Buzsáki G., Kaila K., Raichle M. (2007). Inhibition and brain work. Neuron.

[56] Verma V., Bali A., Singh N., Jaggi A.S. (2015). Implications of sodium hydrogen exchangers in various brain diseases. J. Basic Clin. Physiol. Pharmacol..

[57] Song S., Luo L., Sun B., Sun D. (2020). Roles of glial ion transporters in brain diseases. Glia.

[58] Jung H., Kim S.Y., Canbakis Cecen F.S., Cho Y., Kwon S.K. (2020). Dysfunction of Mitochondrial Ca2+ Regulatory Machineries in Brain Aging and Neurodegenerative Diseases. Front. Cell Dev. Biol..

[59] Bak L.K., Schousboe A., Waagepetersen H.S. (2006). The glutamate/GABA-glutamine cycle: Aspects of transport, neurotransmitter homeostasis and ammonia transfer. J. Neurochem..

[60] Roth F.C., Draguhn A. (2012). GABA metabolism and transport: Effects on synaptic efficacy. Neural. Plast..

[61] Vien T.N., Moss S.J., Davies P.A. (2016). Regulating the Efficacy of Inhibition Through Trafficking of γ-Aminobutyric Acid Type A Receptors. Anesth Analg..

[62] Groc L., Choquet D. (2020). Linking glutamate receptor movements and synapse function. Science.

[63] Ryan R.M., Ingram S.L., Scimemi A. (2021). Regulation of Glutamate, GABA and Dopamine Transporter Uptake, Surface Mobility and Expression. Front. Cell Neurosci..

[64] Ghosh Dastidar S., Das Sharma S., Chakraborty S., Chattarji S., Bhattacharya A., Muddashetty R.S. (2020). Distinct regulation of bioenergetics and translation by group I mGluR and NMDAR. EMBO Rep..

[65] Rodríguez M., Valez V., Cimarra C., Blasina F., Radi R. (2020). Hypoxic-Ischemic Encephalopathy and Mitochondrial Dysfunction: Facts, Unknowns, and Challenges. Antioxid. Redox Signal..

[66] Ladak Z., Garcia E., Yoon J., Landry T., Armstrong E.A., Yager J.Y., Persad S. (2021). Sulforaphane (SFA) protects neuronal cells from oxygen & glucose deprivation (OGD). PLoS ONE.

[67] Gava-Junior G., Roque C., Mendes-Oliveira J., Bernardino A.C., Serrenho I., Pires J.P., Baltazar G. (2020). A Cell Culture Model for Studying the Role of Neuron-Glia Interactions in Ischemia. J. Vis. Exp..

[68] He Z., Hu M., Zha Y.H., Li Z.C., Zhao B., Yu L.L., Yu M., Qian Y. (2014). Piracetam ameliorated oxygen and glucose deprivation-induced injury in rat cortical neurons via inhibition of oxidative stress, excitatory amino acids release and P53/Bax. Cell. Mol. Neurobiol..

[69] Shi L., Zhang J., Wang Y., Hao Q., Chen H., Cheng X. (2020). Sirt1 Regulates Oxidative Stress in Oxygen-Glucose Deprived Hippocampal Neurons. Front. Pediatr..

[70] Wu X., Wang C., Wang J., Zhu M., Yao Y., Liu J. (2021). Hypoxia preconditioning protects neuronal cells against traumatic brain injury through stimulation of glucose transport mediated by HIF-1α/GLUTs signaling pathway in rat. Neurosurg. Rev..

[71] Muzzi L., Hassink G., Levers M., Jansman M., Frega M., Hofmeijer J., van Putten M., le Feber J. (2019). Mild stimulation improves neuronal survival in an in vitro model of the ischemic penumbra. J. Neural Eng..

[72] Ozugur S., Kunz L., Straka H. (2020). Relationship between oxygen consumption and neuronal activity in a defined neural circuit. BMC Biol..

[73] Zhang X., Gaetani M., Chernobrovkin A., Zubarev R.A. (2019). Anticancer Effect of Deuterium Depleted Water—Redox Disbalance Leads to Oxidative Stress. Mol. Cell Proteom..

